# Modeling of Nitrogen Dynamics in an Austrian Alpine Forest Ecosystem on Calcareous Soils: A Scenario-Based Risk Assessment under Changing Environmental Conditions

**DOI:** 10.1100/tsw.2007.9

**Published:** 2007-03-21

**Authors:** Friedl Herman, Stefan Smidt, Klaus Butterbach-Bahl, Michael Englisch, Ernst Gebetsroither, Robert Jandl, Klaus Katzensteiner, Manfred Lexer, Friederike Strebl, Sophie Zechmeister-Boltenstern

**Affiliations:** ^1^ Federal Research and Training Centre for Forests, Natural Hazards and Landscape (BFW Vienna), Vienna, Austria; ^2^ Institute for Meteorology and Climate Research (IMK-IFU), Garmisch-Partenkirchen, Germany; ^3^ Austrian Research Centers, Seibersdorf, Austria; ^4^ University of Natural Resources and Applied Life Sciences, Vienna, Austria

**Keywords:** nitrogen dynamics, Austrian limestone alps, risk assessment, global change, nitrate leaching, greenhouse gas emissions

## Abstract

We modeled the behavior of an Austrian alpine forest ecosystem on calcareous soils under changing climate and atmospheric nitrogen deposition scenarios. The change of nitrate leaching, emission rates of nitrogen compounds, and forest productivity were calculated using four process-oriented models for the periods 1998–2002 and 2048–2052. Each model reflects with high detail a segment of the ecosystem: PnET-N-DNDC (photosynthesis-evapotranspiration-nitrification-denitrification-decomposition; shortterm nitrogen cycling), BROOK90 (water balance for small and homogenous forest watersheds), HYDRUS (water flux in complex and heterogenous soils), and PICUS v1.3 (forest productivity). The nitrogen balance model (NBM) combines the individual results into a comprehensive picture and extends the specific values beyond the limits of the individual models. The evaluation of the findings was outlined with TRACE, a model enabling a long-term prognosis of nitrogen cycling in annual time steps.

Temperature increase and nitrogen input are influenced by various components and processes of the forest ecosystem. An increase of the temperature of 2.5°C led to an enhancement of the N_2_O emission rates and affected the mineralization and the nitrification rates with the consequence of increased nitrate leaching into the subsoil. Enhanced nitrogen input also showed notable effects on nitrate leaching.

## INTRODUCTION

High rates of nitrogen deposition are still an unresolved problem for Central European forests. Ambient concentrations of NOx in Austria frequently exceed threshold values and the Critical Loads of the World Health Organization[1,2,3]. After a reduction phase during the 1980s, the NOx emissions have again increased significantly in Austria since 1995. Exceedances of the EU-limit of 30 µg NOx $m^{-3}$ (annual mean value, which is also the legal standard in Austria) occur frequently[4]. The risk of eutrophication was assessed for the North Tyrolean Limestone Alps (NTLA), an area of 400.000 ha with a Steady State Mass Balance Model; the Critical Loads calculated for forests at the valley slopes along highways were low, which means a high sensitivity regarding nitrogen input[5]. Occult deposition (fogwater) contributes substantially to the total deposition especially in high elevations[6]. In summary, nitrogen input is a potential risk for forest ecosystem stability at all elevations.

The global rise of temperature since 1900 (approx. 0.8°C) and especially since 1980 is also evident in Austria[7], where an increase of 1.5°C was observed since 1900. This will lead to an enhanced risk for the main Austrian tree species, *Picea abies*[8]. It is not clear how existing forests will cope with this warming.

The above-mentioned facts were the reason for considering the effects of increasing temperature and different nitrogen inputs on the formation of $N_2O$ emissions, nitrate leaching, and forest productivity. Based on various model approaches, it was possible to evaluate the processes for a time period of 50 years[9].

## MATERIALS AND METHODS

### Study Site

The study site located in the NTLA is an intensively monitored research plot of 0.3 ha. The stand consists mainly of 125-year-old *P. abies* (L.) Karst. with admixed *Fagus sylvatica* (L.). The coordinates of the research plot are 11° 38’ 21” East and 47° 34’ 50” North, the elevation is 895 m a.s.l.[10]. This site is typical for the area of the NTLA, considering the bedrock, climate, tree composition, and vegetation.

### Database

Many years of monitoring activities and research projects provide a time series of key elements of the nitrogen cycle: input (canopy uptake; open-field deposition including wet, dry, and occult deposition as well as throughfall deposition), pools (above-ground biomass, organic and inorganic nitrogen in the soil and soil water[11]), internal fluxes (litterfall, surface runoff, soil water and nitrate relocation into microbial biomass, net mineralization rate, plant available nitrogen), and output as $N_2O$ emission rates[12] and nitrate fluxes into the subsoil[13,14]. All these data were included into the respective models.

### Scenarios

The scenarios were:

- SC1 — Current temperature and actual nitrogen deposition (18 kg N $ha^{-1}$ $year^{-1}$)
- SC2 — Warmer climate (increase of 2.5°C of the mean annual temperature) and N deposition reduced by 50% (9 kg N $ha^{-1}$ $year^{-1}$)
- SC3 — Warmer climate and doubled N deposition (36 kg N $ha^{-1}$ $year^{-1}$)

- SC4 — Warmer climate and nitrogen deposition as actual

Two time periods 1998–2002 and 2048–2052 have been assessed for comparative purposes.

## Models

The key element of the research approach was the combined application of six models to analyze system behavior. The models used were:

- PnET-N-DNDC (photosynthesis-evapotranspiration-nitrification-denitrification-decomposition) is an ecosystem model for the simulation of the carbon and nitrogen turnover (short-term nitrogen cycling) in temperate forests, focusing on the formation of $N_2O$, $N_2$, and NO in soils[15,16]. It works on the ecosystem level and emphasizes vegetation physiology, biogeochemistry, and hydrology and simulates photosynthesis as a function of the nitrogen content of needles, transpiration, water vapor deficit, stomatal conductivity, and availability of the soil water. It was optimized for the exact calculation of the emission of nitrogen compounds out of the soil by explicitly simulating the involved key processes such as nitrification and denitrification.
- BROOK90 is a hydrologic model that simulates precipitation, evapotranspiration, and runoff (water balance) for small and homogenous forest watersheds[17]. It requires only a few meteorological parameters. The soil properties are described using the relation between soil water content, matrix potential, and hydraulic conductivity.
- HYDRUS simulates the horizontal water flux in complex and heterogeneous soils[18,19]. The linkage with the average $NO_3$-N concentration in the soil solution provides the estimate of the annual aqueous nitrogen output.
- PICUS v1.3 models the forest productivity and the primary net production of a forest stand caused by environmental changes considering population dynamics[9,20]. The development of individual trees within a cohort is simulated depending on light, temperature, water, and nutrient supply and is integrated in a physiology-based stand model[9].

Each of the individual models reflect with high detail a segment of the ecosystem, e.g., PICUS simulates biomass growth without taking into account soil processes, while PnET-N-DNDC is focused to soil microbial processes, but uses a very basic approach to take into account tree growth. The consolidation and evaluation of the results was performed with two models:

- The nitrogen balance model (NBM[21]) combines the results of the individual models to form a most comprehensive as possible picture of the nitrogen balance for a forest ecosystem in the NTLA. NBM contains all relevant nitrogen pools, inputs and outputs, and extends the specific values beyond the limits of the individual models. A balancing of all nitrogen pools and fluxes permits a check of the credibility of the individual model findings, as discrepancies at points of intersections of the models became apparent. The dispatcher “mineralized nitrogen pool” separates the various fluxes into the different compartments (Fig. 1). NBM gives a comprehensive picture of the nitrogen fluxes between ecosystem compartments.
- With the process-oriented biogeochemical model TRACE[22], the description of the carbon, nitrogen, and water fluxes and pools were evaluated. The model enables a long-term prognosis in annual time steps.

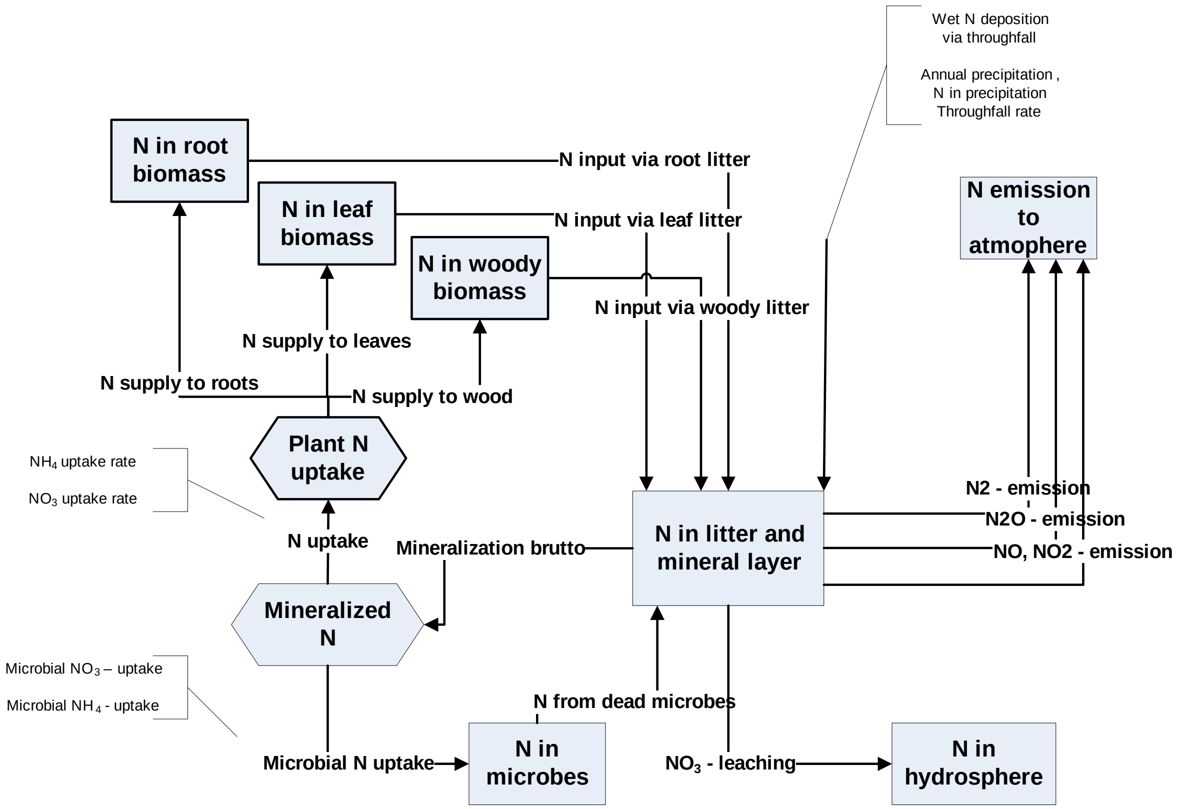

**FIGURE 1**. Pools, fluxes, and dispatchers in the NBM.

## RESULTS

Table 1 shows the results regarding emission rates from the soil, and the microbial turnover processes (SC1-SC4) obtained with PnET-N-DNDC as well as the quantities of the above- and below-ground tree biomass modeled with PICUS (SC1 and SC4). The modeled and measured values were in acceptable agreement. The influence of increasing temperature and nitrogen is obvious. Internal inconsistencies of the models were identified by means of measured data.

One of the key findings of NBM, the nitrate leaching, is outlined in Table 2. A comparison with the results gained through PnET-N-DNDC, BROOK90/HYDRUS, and TRACE is also provided. While BROOK90 and HYDRUS overestimated nitrate leaching, the results of NBM corresponded well with the measured values. TRACE corroborates the findings of NBM. The most important results were as follows:

**Increasing temperature led** to

- Enhanced $N_2O$ emissions.
- A significant increase of the mineralization and nitrification rates (nitrogen deposition caused these processes to a minor degree).
- More nitrate formation in the soil and therefore an enhanced risk of nitrate leaching into the subsoil and the aquifer.
- Increased nitrogen in the woody and root biomass.
- The simulation of the increment of the stemwood biomass over the next 50 years gave a mean annual increment of 6.2 $m^3$ p.a. under present climatic conditions. Elevated temperature led to a decrease to 5.8 $m^3$ p.a. due to an elevated increase in tree mortality.

**TABLE 1**
**Results of PnET-N-DNDC and PICUS for the Main Parameters within the Scenarios**

| **Atmospheric Deposition** kg N $ha^{-1}$ $a^{-1}$ | **SC1** 18 kg N $ha^{-1}$ $a^{-1}$ (Current Temperature) | **SC2** 9 kg N $ha^{-1}$ $a^{-1}$ (+2.5°C) | **SC4** 18 kg N $ha^{-1}$ $a^{-1}$ (+2.5°C) | **SC3** 36 kg N $ha^{-1}$ $a^{-1}$ (+2.5°C) |
|---|---|---|---|---|
| **PnET-N-DNDC** | | | | |
| $N_2$ emission | 15.6 | 16.1 | 15.6 | 15.9 |
| $N_2O$ emission[1] | 1.2 | 1.6 | 1.7 | 2.0 |
| NO emission[2] | 0.07 | 0.06 | 0.06 | 0.07 |
| Total gaseous nitrogen loss | 16.9 | 17.8 | 17.4 | 18.0 |
| N release from dead microbes | 218 | 244 | 248 | 258 |
| Cumulative $NO_3$ uptake by microbes | 174 | 201 | 206 | 221 |
| Cumulative $NH_4$ uptake by microbes | 35 | 39 | 39 | 39 |
| Gross N mineralization | 242 | 292 | 296 | 306 |
| Gross nitrification | 196 | — | — | — |
| Microbial biomass N (kg $ha^{-1}$) | 516 | 520 | 526 | 544 |
| **PICUS** | | | | |
| N in the root biomass | 128 | — | 178 | — |
| N in the needle biomass[3] | 155 | — | 146 | — |
| N in the woody biomass[4] | 459 | — | 634 | — |
| N input through root litter | 13 | — | 19 | — |
| N input through needle litter[5] | 22 | — | 21 | — |
| N input through woody litter | 11 | — | 14 | — |

Field data[11]: [1]0.9 kg $N_2O$-N $ha^{-1}$ $a^{-1}$; [2]0.03 kg NO-N $ha^{-1}$ $a^{-1}$; [3]176 kg N $ha^{-1}$; [4]397 kg N $ha^{-1}$; [5]25.1 kg N $ha^{-1}$.

**TABLE 2**
**Nitrate Leaching in Accordance with the Results from NBM, BROOK90/HYDRUS, and TRACE**

| **Nitrate Leaching into the Subsoil** kg $NO_3$-N $ha^{-1}a^{-1}$ | **SC1** Actual N Deposition 18 kg N $ha^{-1}$ $a^{-1}$ (Mean Temperature 1998–2002) | **SC2** 9 kg N $ha^{-1}$ $a^{-1}$ (+2.5°C) | **SC4** 18 kg N $ha^{-1}$ $a^{-1}$ (+2.5°C) | **SC3** 36 kg N $ha^{-1}$ $a^{-1}$ (+2.5°C) |
|---|---|---|---|---|
| PnET-N-DNDC | 1.1 | 3.0 | 3.3 | 5.1 |
| UNSATCHEM / HYDRUS1D 1999, 2000[14] | 4–10 | | | |
| BROOK90 / HYDRUS | 14.5 | — | 15.6 | — |
| TRACE results for 2000 | 5.3 | 7.6 | 10.4 | 16.0 |
| TRACE results for 2050 | 10.9 | 11.5 | 16.2 | 25.3 |
| NBM | 3 | 6 | 11 | 24 |
| Measured 1999, 2000[13] | 15.9; 7.9 | — | — | — |

**Increasing nitrogen input** led only to a slightly increased formation of $N_2O$, but to an escalated nitrate leaching already under the current nitrogen deposition, especially if the nitrogen deposition was doubled (Table 2).

## CONCLUSIONS

- The combination of different models yielded improved predictions in comparison to the individual models. All relevant processes of the nitrogen balance were captured. The results of the individual models were combined and validated with the results of the open-field measurements and literature references.
- Forest soils may contribute significantly to the $N_2O$ emissions especially under increasing temperature (up to 2 kg N $ha^{-1}$ $year^{-1}$). The anthropogenic emissions, related to the total area of Austria, are presently 0.7 kg N $ha^{-1}$ $year^{-1}$[23].
- Increasing temperature led to enhanced nitrogen mineralization rates. This is in accordance with field experiments over 18 months at the study site showing strong temperature dependency of net nitrogen mineralization and net nitrification[12]. The model scenarios reflecting a temperature increase also displayed a distinct rise of the gross mineralization rate. This supports the importance of mineralization and nitrification as the key nitrogen mobilization processes contributing to nitrate leaching, the cardinal symptom of nitrogen saturation.
- Enhanced nitrate leaching into the groundwater under changing environmental conditions has been verified.
- A combination of the risk factors "elevated temperature and nitrogen inputs" has a distinct negative effect on forest ecosystems.
